# New anatomical data on the growing C4 vertebra and its three ossification centers in human fetuses

**DOI:** 10.1007/s00276-012-1022-z

**Published:** 2012-09-18

**Authors:** Mariusz Baumgart, Michał Szpinda, Anna Szpinda

**Affiliations:** Department of Normal Anatomy, The Ludwik Rydygier Collegium Medicum in Bydgoszcz, The Nicolaus Copernicus University in Toruń, Karłowicza 24 Street, 85-092 Bydgoszcz, Poland

**Keywords:** Typical cervical vertebra, Ossification center, Dimensions, CT examination, Digital image analysis, Skeletodysplasias, Human fetuses

## Abstract

**Purpose:**

Detailed knowledge on the normative growth of the spine is of great relevance in the prenatal diagnosis of its abnormalities. The present study was conducted to compile age-specific reference data for vertebra C4 and its three ossification centers in human fetuses.

**Materials and methods:**

With the use of CT (Biograph mCT), digital image analysis (Osirix 3.9) and statistical analysis (Wilcoxon signed-rank test, Kolmogorov–Smirnov test, Levene’s test, Student’s *t* test, one-way ANOVA, post hoc RIR Tukey test, linear and nonlinear regression analysis), the normative growth of vertebra C4 and its three ossification centers in 55 spontaneously aborted human fetuses (27 males, 28 females) aged 17–30 weeks was examined.

**Results:**

Significant differences in neither sex nor laterality were found. The height and transverse and sagittal diameters of the C4 vertebral body increased logarithmically as: *y* = −3.866 + 2.225 × ln(Age) ± 0.238 (*R* ^2^ = 0.69), *y* = −7.077 + 3.547 × ln(Age) ± 0.356 (*R* ^2^ = 0.72) and *y* = −3.886 + 2.272 × ln(Age) ± 0.222 (*R* ^2^ = 0.73), respectively. The C4 vertebral body grew linearly in cross-sectional area as *y* = −7.205 + 0.812 × Age ± 1.668 (*R* ^2^ = 0.76) and four-degree polynomially in volume as *y* = 14.108 + 0.00007 × Age^4^ ± 6.289 (*R* ^2^ = 0.83). The transverse and sagittal diameters, cross-sectional area and volume of the ossification center of the C4 vertebral body generated the following functions: *y* = −8.836 + 3.708 × ln(Age) ± 0.334 (*R* ^2^ = 0.76), *y* = −7.748 + 3.240 × ln(Age) ± 0.237 (*R* ^2^ = 0.83), *y* = −4.690 + 0.437 × Age ± 1.172 (*R* ^2^ = 0.63) and *y* = −5.917 + 0.582 × Age ± 1.157 (*R* ^2^ = 0.77), respectively. The ossification center-to-vertebral body volume ratio gradually declined with age. On the right and left, the neural ossification centers showed the following growth: *y* = −19.601 + 8.018 × ln(Age) ± 0.369 (*R* ^2^ = 0.92) and *y* = −15.804 + 6.912 × ln(Age) ± 0.471 (*R* ^2^ = 0.85) for length, *y* = −5.806 + 2.587 × ln(Age) ± 0.146 (*R* ^2^ = 0.88) and *y* = −5.621 + 2.519 × ln(Age) ± 0.146 (*R* ^2^ = 0.88) for width, *y* = −9.188 + 0.856 × Age ± 2.174 (*R* ^2^ = 0.67) and *y* = −7.570 + 0.768 × Age ± 2.200 (*R* ^2^ = 0.60) for cross-sectional area, and *y* = −13.802 + 1.222 × Age ± 1.872 (*R* ^2^ = 0.84) and *y* = −11.038 + 1.061 × Age ± 1.964 (*R* ^2^ = 0.80) for volume, respectively.

**Conclusions:**

The morphometric parameters of vertebra C4 and its three ossification centers show no sex differences. The C4 vertebral body increases logarithmically in height and both sagittal and transverse diameters, linearly in cross-sectional area, and four-degree polynomially in volume. The three ossification centers of vertebra C4 grow logarithmically in both transverse and sagittal diameters, and linearly in both cross-sectional area and volume. The age-specific reference intervals for evolving vertebra C4 may be useful in the prenatal diagnosis of congenital spinal defects.

## Introduction

Advances in ultrasound devices have facilitated the assessment of most fetal structures and improved the prenatal diagnostics [1, 16, 25, 30]. Both CT and MRI examinations of the vertebral column are often superior to ultrasonography for evaluation of spinal anomalies [6, 8, 12, 15, 19, 21]. Detailed knowledge on the normative growth of the spine is relevant for diagnosing its abnormalities [12, 15, 23, 30, 33] and skeletal dysplasias [29].

The typical cervical vertebra is approximately 1/2 and 2/3 of the height of the lumbar and thoracic vertebrae, respectively [2]. Any vertebra ossifies from the three primary ossification centers, one existing in the vertebral body and one occurring in each neural process [2–4, 22]. Developmental pathways of the appearance of ossification centers for the neural processes and vertebral bodies evolve completely independently of each other in a definite topographical sequence [2]. Therefore, ossification of vertebral bodies starts with the thoracolumbar junction to proceed bi-directionally in both cranial and caudal directions [24]. The three ossification pathways of the neural processes have been postulated: firstly, originating in the thoracolumbar, cervico-thoracic, and superior cervical regions [4]; secondly, originating in the mid-thoracic spinal region [22]; and thirdly, originating in the superior cervical region [3].

To date, little has been known in the medical literature on morphometric values for cervical vertebrae in human fetuses [2, 24, 28], and the quantitative analysis of vertebral ossification centers has not been reported yet. Among other cervical vertebrae, we have specifically looked at the C4 vertebra, being a typical mid-cervical one. Its growth patterns will be useful in further understanding the development of adjacent vertebrae, in both proximal and distal directions. For this reason, to supplement fragmentary information about the dimensions of the C4 vertebra and its ossification centers, our objectives were set to examine the following:

- age-specific reference intervals for height, transverse and sagittal diameters, cross-sectional area, and volume of its vertebral body;
- age-specific reference intervals for transverse and sagittal diameters, cross-sectional area, and volume of its three ossification centers;
- the best-fit growth curves for each parameter examined;
- the relative growth of the ossification center within the vertebral body (the ossification center-to-vertebral body volume ratio).

## Materials and methods

**Fig. 1:**
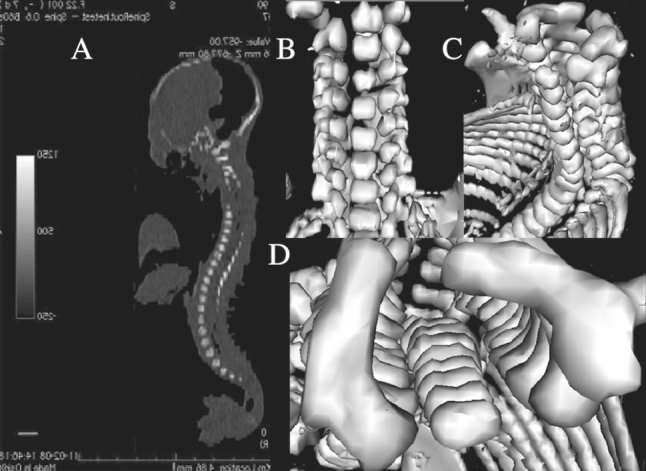
CT of a female fetus aged 25 weeks recorded in DICOM formats (**a**) and assessed by Osirix 3.9 in frontal (**b**), lateral (**c**), and horizontal (**d**) planes

The present study included 55 ethnically homogenous human fetuses (27 males, 28 females) aged 17–30 weeks, of Caucasian racial origin (Table 1), which had been derived from spontaneous abortions or stillbirths in the years 1989–2001 because of placental insufficiency. Gestational ages were determined from measurements of the fetal crown–rump length [14]. No attempt was done to encourage fetal donation. The use of the fetuses for research was accepted by the University Research Ethics Committee (KB 275/2011). None of the fetuses demonstrated visible malformations. For preservation, all specimens were immersed in 10 % neutral buffered formalin solution. The fetuses underwent CT examinations with the reconstructed slice width option of 0.4 mm and 128 slices were acquired simultaneously by Biograph mCT (Siemens). The CT scans obtained were recorded in DICOM formats (Fig. 1a), with possibility to create three-dimensional reconstructions and the morphometric analysis of structures examined. Measurements of the vertebral column could be performed only after identifying vertebra C4. Next, DICOM formats were assessed using digital image analysis of Osirix 3.9 (Fig. 1b–d) with estimating linear (sagittal and transverse diameters, height, length, width), two-dimensional (cross-sectional area), and three-dimensional (volume) parameters of vertebra C4. The contouring procedure of each C4 vertebral body and the three ossification centers was outlined with a cursor and recorded.

**Table 1:** Age, number, and sex of the fetuses studied

Gestational age (weeks)	Crown–rump length (mm)				Number	Sex	
	Mean	SD	Min	Max		Male	Female
17	115.00		115.00	115.00	1	0	1
18	133.33	5.77	130.00	140.00	3	1	2
19	149.50	3.82	143.00	154.00	8	3	5
20	161.00	2.71	159.00	165.00	4	2	2
21	174.75	2.87	171.00	178.00	4	3	1
22	185.00	1.41	183.00	186.00	4	1	3
23	197.60	2.61	195.00	202.00	5	2	3
24	208.67	3.81	204.00	213.00	9	5	4
25	214.00		214.00	214.00	1	0	1
26	229.00	5.66	225.00	233.00	2	1	1
27	239.17	3.75	235.00	241.00	6	6	0
28	249.50	0.71	249.00	250.00	2	0	2
29	253.00	0.00	253.00	253.00	2	0	2
30	263.25	1.26	262.00	265.00	4	3	1
Total					55	27	28

The five following parameters of the C4 vertebral body were evaluated for each fetus:

1. height (in mm), corresponding to the distance between the superior and inferior borderlines of the vertebral body (in sagittal projection),

2. transverse diameter (in mm), corresponding to the distance between the left and right borderlines of the vertebral body (in transverse projection),

3. sagittal diameter (in mm), corresponding to the distance between the anterior and posterior borderlines of the vertebral body (in sagittal projection),

4. cross-sectional area (in mm^2^), traced around the vertebral body (in transverse projection), and

5. volume (in mm^3^).

In addition, the 12 following parameters of the three ossifications centers were assessed for each fetus: within the vertebral body (6–9):

6. transverse diameter (in mm), corresponding to the distance between the left and right borderlines of the ossification center (in transverse projection),

7. sagittal diameter (in mm), corresponding to the distance between the anterior and posterior borderlines of the ossification center (in sagittal projection),

8. cross-sectional area (in mm^2^), traced around the ossification center (in transverse projection),

9. volume (in mm^3^), and within the right and left neural processes (10–17):

10, 11. right and left lengths (in mm), corresponding to the distance between the anterior and posterior borderlines of the ossification center (in transverse projection),

12, 13. right and left widths (in mm), corresponding to the distance between the left and right borderlines of the ossification center (in transverse projection),

14, 15. right and left cross-sectional areas (in mm^2^), traced around the ossification center (in transverse projection),

16, 17. right and left volumes (in mm^3^).

In a continuous effort to minimize measurements and observer bias, all the measurements were performed by one researcher (M.B). Each measurement was repeated three times under the same conditions, but at different times, and the mean of the three was finally used. The findings obtained were subjected to statistical analysis. The intra-observer variation was assessed by the Wilcoxon signed-rank test. All the parameters examined were plotted versus gestational age to construct their growth dynamics. The relative growth, both at the vertebral body and its ossification center, was expressed as the sagittal-to-transverse diameter ratios and the ossification center-to-vertebral body volume ratio. The data obtained was checked for normality of distribution using the Kolmogorov–Smirnov test and homogeneity of variance with the use of Levene’s test. As a consequence of the statistical analysis, Student’s *t* test was used to examine the impact of sex on the values obtained. In order to examine sex differences, we tested possible differences between the five following age groups: 17–19, 20–22, 23–25, 26–28, and 29–30 weeks. Next, we checked sex differences for the whole examined group, without taking into account gestational age. To check whether variables changed significantly with age, the one-way ANOVA test and the post hoc RIR Tukey test were used for the five age groups mentioned above. Linear and nonlinear regression analysis was used to derive the best-fit curve for each parameter studied versus gestational age, with estimating coefficients of determination (*R* ^2^) between each parameter and fetal age.

## Results

**Fig. 2:**
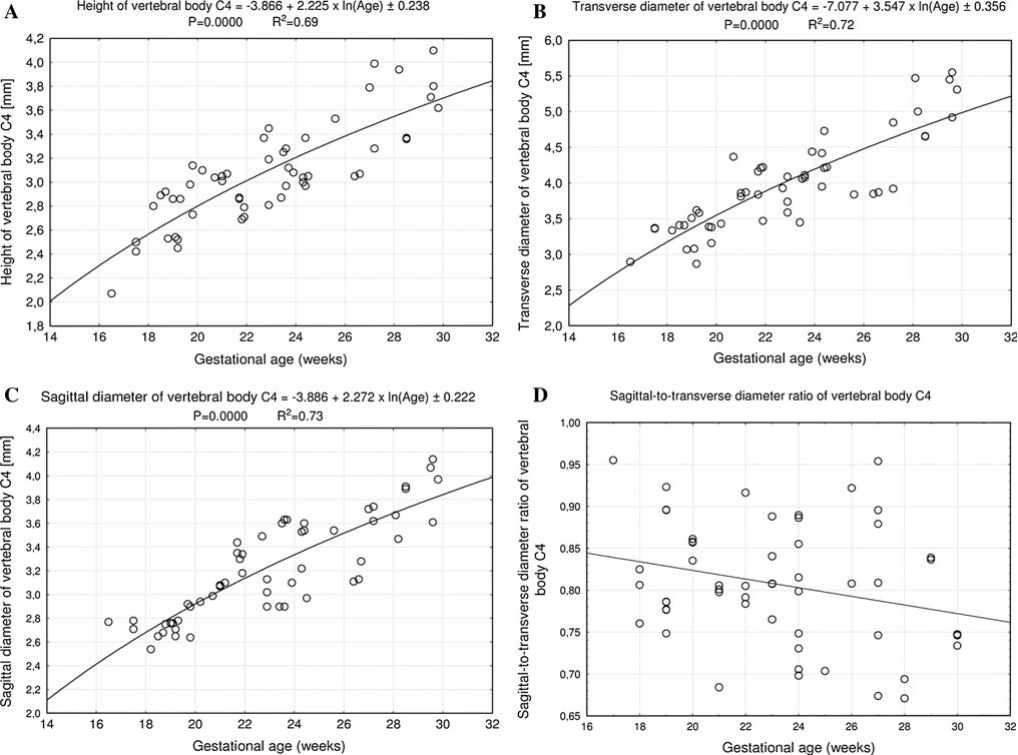
Regression lines for height (**a**), transverse diameter (**b**), sagittal diameter (**c**), and sagittal-to-transverse diameter ratio (**d**) of the vertebral body C4

**Fig. 3:**
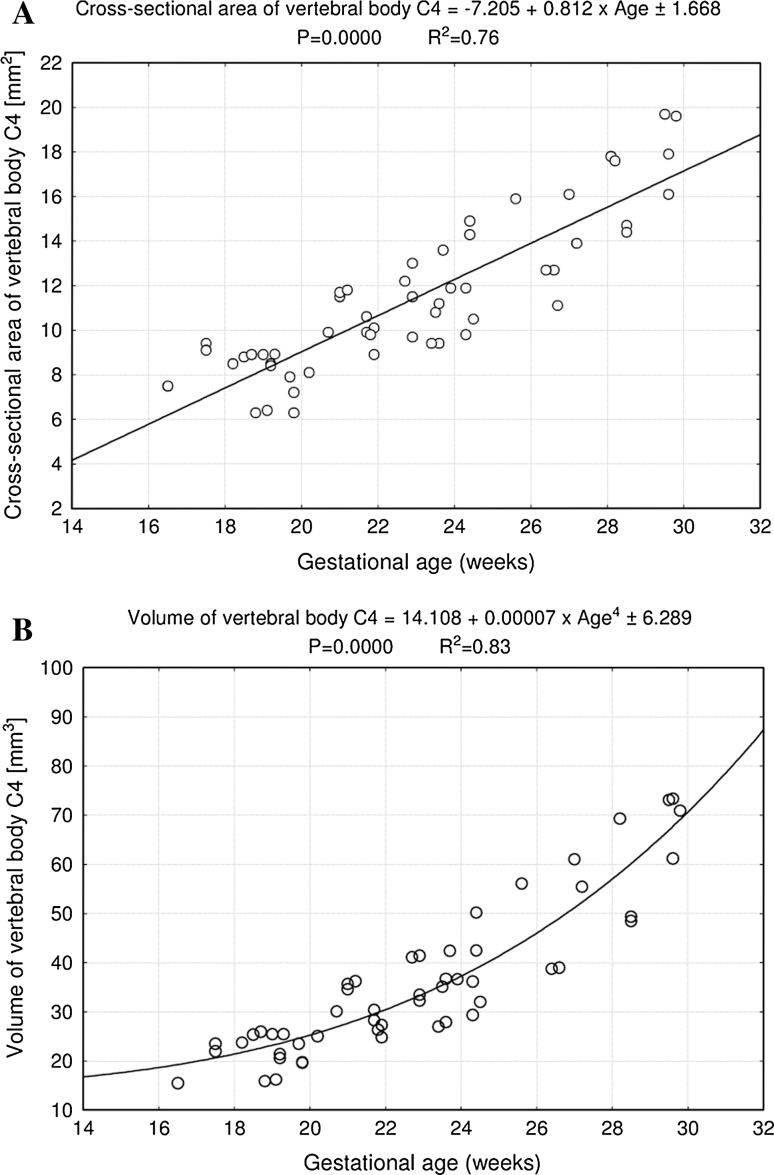
Regression lines for cross-sectional area (**a**) and volume (**b**) of the vertebral body C4

No statistically significant differences were observed in assessing intra-observer reproducibility of the spinal measurements. In addition, no significant difference was found in the values of the parameters studied according to sex, so the morphometric values for vertebra C4 (Table 2) and its ossification centers (Tables 3, 4) have been summarized for both sexes. By contrast, a statistically significant (*P* = 0.0000) increase in values of all the measurements was accompanied by advancing gestational age. The numerical data correlated to gestational age presented differentiated growth dynamics, expressed by specific best-fit growth curves (Figs. 2, 3 and 5–8).

**Table 2:** Morphometric parameters of the C4 vertebral body

Age (weeks)	*n*	Height (mm)		Transverse diameter (mm)		Sagittal diameter (mm)		Cross-sectional area (mm^2^)		Volume (mm^3^)	
		Mean	SD	Mean	SD	Mean	SD	Mean	SD	Mean	SD
17	1	2.07		2.90		2.77		7.50		15.53	
18	3	2.57	0.20	3.36	0.02	2.68	0.12	9.00	0.46	23.11	0.95
19	8	2.70	0.20	3.32	0.28	2.72	0.05	8.14	1.12	22.07	4.21
		↓ (*P* < 0.05)		↓ (*P* < 0.01)		↓ (*P* < 0.05)		↓ (*P* < 0.01)		↓ (*P* < 0.001)	
20	4	2.99	0.18	3.34	0.12	2.85	0.14	7.38	0.81	22.02	2.74
21	4	3.04	0.03	3.98	0.26	3.06	0.05	11.23	0.89	34.16	2.79
22	4	2.76	0.08	4.02	0.36	3.29	0.08	9.68	0.53	26.72	1.49
		↓ (*P* < 0.05)		↓ (*P* < 0.01)		↓ (*P* < 0.05)		↓ (*P* < 0.05)		↓ (*P* < 0.01)	
23	5	3.14	0.29	3.76	0.26	3.09	0.24	11.16	1.57	35.07	6.19
24	9	3.12	0.15	4.33	0.34	3.42	0.27	11.98	1.94	38.84	8.94
25	1	3.05		4.22		2.97		10.50		32.03	
		↓ (*P* < 0.05)		↓ (*P* < 0.01)		↓ (*P* < 0.01)		↓ (*P* < 0.01)		↓ (*P* < 0.01)	
26	2	3.29	0.34	3.85	0.01	3.33	0.30	13.80	2.26	49.44	19.45
27	6	3.28	0.52	4.29	0.73	3.49	0.25	11.90	3.12	36.59	13.24
28	2	4.06	0.16	5.24	0.33	3.57	0.14	17.70	0.14	71.79	3.45
		↓ (*P* < 0.05)		↓ (*P* < 0.05)		↓ (*P* < 0.01)		↓ (*P* < 0.001)		↓ (*P* < 0.01)	
29	2	3.37	0.01	4.66	0.01	3.90	0.01	14.55	0.21	48.96	0.61
30	4	3.81	0.21	5.31	0.28	3.95	0.24	18.33	1.70	72.43	9.46

**Table 3:** Morphometric parameters of the ossification center of vertebra C4

Age (weeks)	*n*	Vertebral body							
		Transverse diameter (mm)		Sagittal diameter (mm)		Cross-sectional area (mm^2^)		Volume (mm^3^)	
		Mean	SD	Mean	SD	Mean	SD	Mean	SD
17	1	1.60		1.32		3.70		4.67	
18	3	1.91	0.07	1.51	0.07	3.23	0.21	4.50	0.64
19	8	1.89	0.18	1.70	0.06	2.81	0.42	4.61	0.94
		↓ (*P* < 0.001)		↓ (*P* < 0.001)		↓ (*P* < 0.001)		↓ (*P* < 0.001)	
20	4	2.30	0.35	1.86	0.31	3.93	0.75	5.23	0.94
21	4	2.61	0.20	2.50	0.08	5.43	0.34	7.61	0.21
22	4	2.81	0.28	2.32	0.15	5.10	0.45	7.41	0.23
		↓ (*P* < 0.01)		↓ (*P* < 0.05)		↓ (*P* < 0.05)		↓ (*P* < 0.001)	
23	5	2.63	0.31	2.29	0.15	4.48	1.19	6.52	1.55
24	9	3.09	0.39	2.52	0.28	6.62	1.06	9.14	1.46
25	1	3.18		2.30		5.80		8.28	
		↓ (*P* < 0.01)		↓ (*P* < 0.01)		↓ (*P* < 0.001)		↓ (*P* < 0.01)	
26	2	3.15	0.68	2.99	0.10	6.75	3.18	9.78	0.88
27	6	3.18	0.49	2.81	0.14	5.70	1.83	7.45	1.91
28	2	4.00	0.01	2.74	0.01	8.05	1.06	9.90	0.42
		↓ (*P* < 0.05)		↓ (*P* < 0.001)		↓ (*P* < 0.01)		↓ (*P* < 0.001)	
29	2	3.25	0.03	3.16	0.01	8.20	0.14	10.60	0.14
30	4	3.74	0.44	3.37	0.12	10.23	3.14	13.45	3.20

**Table 4:** Morphometric parameters of ossification centers of neural processes of vertebra C4

Age (weeks)	*n*	Right neural arch								Left neural arch							
		Length (mm)		Width (mm)		Cross-sectional area (mm^2^)		Volume (mm^3^)		Length (mm)		Width (mm)		Cross-sectional area (mm^2^)		Volume (mm^3^)	
		Mean	SD	Mean	SD	Mean	SD	Mean	SD	Mean	SD	Mean	SD	Mean	SD	Mean	SD
17	1	3.40		1.66		6.40		8.37		3.49		1.62		4.60		6.17	
18	3	3.52	0.23	1.63	0.06	8.50	1.82	8.45	1.03	3.83	0.67	1.63	0.05	8.80	0.70	8.30	0.95
19	8	3.88	0.08	1.74	0.07	5.92	0.78	9.01	0.84	4.63	0.11	1.74	0.08	6.63	1.67	8.83	1.57
		↓		↓		↓		↓		↓		↓		↓		↓	
		(*P* < 0.001)		(*P* < 0.01)		(*P* < 0.01)		(*P* < 0.01)		(*P* < 0.01)		(*P* < 0.01)		(*P* < 0.01)		(*P* < 0.05)	
20	4	4.64	0.27	1.88	0.08	6.85	0.79	8.65	1.47	4.82	0.43	1.83	0.09	5.68	0.79	8.68	1.51
21	4	4.58	0.44	2.04	0.02	8.58	1.85	11.77	2.27	5.17	0.82	2.02	0.04	6.15	0.51	9.45	0.13
22	4	5.35	0.35	2.21	0.16	8.48	0.22	13.65	1.58	5.62	0.56	2.18	0.14	9.78	1.44	13.60	2.60
		↓		↓		↓		↓		↓		↓		↓		↓	
		(*P* < 0.01)		(*P* < 0.01)		(*P* < 0.001)		(*P* < 0.01)		(*P* < 0.05)		(*P* < 0.01)		(*P* < 0.001)		(*P* < 0.001)	
23	5	5.40	0.25	2.28	0.20	11.58	2.70	15.20	2.65	6.37	0.28	2.25	0.20	11.68	1.82	15.46	3.05
24	9	5.73	0.55	2.56	0.18	12.66	2.26	16.74	2.97	6.10	0.35	2.54	0.17	12.18	1.73	16.46	2.65
25	1	6.29		2.71		11.90		15.50		5.50		2.69		11.20		15.00	
		↓		↓		↓		↓		↓		↓		↓		↓	
		(*P* < 0.01)		(*P* < 0.05)		(*P* < 0.001)		(*P* < 0.001)		(*P* < 0.001)		(*P* < 0.05)		(*P* < 0.01)		(*P* < 0.001)	
26	2	6.91	0.11	2.66	0.01	13.70	3.39	18.40	1.56	6.81	0.54	2.62	0.00	12.05	4.31	16.65	2.19
27	6	5.63	2.08	2.56	0.28	12.67	4.28	17.03	3.62	5.75	1.82	2.50	0.26	10.53	3.09	14.93	3.47
28	2	7.07	0.24	2.97	0.11	16.30	3.68	23.90	5.23	7.78	0.42	2.95	0.11	16.15	1.34	22.15	4.45
		↓		↓		↓		↓		↓		↓		↓		↓	
		(*P* < 0.05)		(*P* < 0.05)		(*P* < 0.01)		(*P* < 0.01)		(*P* < 0.05)		(*P* < 0.05)		(*P* < 0.05)		(*P* < 0.01)	
29	2	7.55	0.01	2.62	0.01	16.05	0.21	20.55	0.07	7.38	0.01	2.59	0.00	15.20	0.14	18.05	0.07
30	4	7.55	0.32	2.67	0.27	18.05	7.36	21.53	3.15	7.38	0.30	2.59	0.32	15.03	5.60	19.83	2.44

The size of the C4 vertebral body has been shown in Table 2. The values of the vertebral body height rose from 2.07 to 3.81 ± 0.21 mm for fetuses aged 17 and 30 weeks, respectively. With advancing gestational age, an increase in height (Fig. 2a) followed logarithmically as *y* = −3.866 + 2.225 × ln(Age) ± 0.238 (*R* ^2^ = 0.69). Between ages of 17 and 30 weeks, the transverse diameter of the vertebral body (Fig. 2b) attained the values from 2.90 to 5.31 ± 0.28 mm, in accordance with the logarithmic function: *y* = −7.077 + 3.547 × ln(Age) ± 0.356 (*R* ^2^ = 0.72). During the duration of the study period, the values of sagittal diameter of the vertebral body (Fig. 2c) increased logarithmically from 2.77 to 3.95 ± 0.24 mm, following the formula: *y* = −3.886 + 2.272 × ln(Age) ± 0.222 (*R* ^2^ = 0.73). Consequently, at ages of 17 and 30 weeks, the growth velocities (mm per week) for height and transverse and sagittal diameters of the vertebral body gradually declined with advancing fetal age (*P* < 0.01), from 0.13 to 0.08 mm, 0.20 to 0.22 mm, and 0.13 to 0.08 mm, respectively. The relative growth of the C4 vertebral body was not proportionate, since the transverse diameter grew much faster than the sagittal diameter. This was expressed by the decrement of the sagittal-to-transverse diameter ratio (Fig. 2d) from 0.84 ± 0.07 to 0.77 ± 0.06 (*P* < 0.01). The values of cross-sectional area of the vertebral body (Fig. 3a) ranged from 7.50 to 18.33 ± 1.70 mm^2^ in fetuses aged 17 and 30 weeks respectively, and generated the linear function *y* = −7.205 + 0.812 × Age ± 1.668 (*R* ^2^ = 0.76). During that time the volumetric growth of the vertebral body (Fig. 3b), from 15.53 to 72.43 ± 9.46 mm^3^, modeled the four-degree polynomial regression *y* = 14.108 + 0.00007 × Age^4^ ± 6.289 (*R* ^2^ = 0.83).

**Fig. 4:**
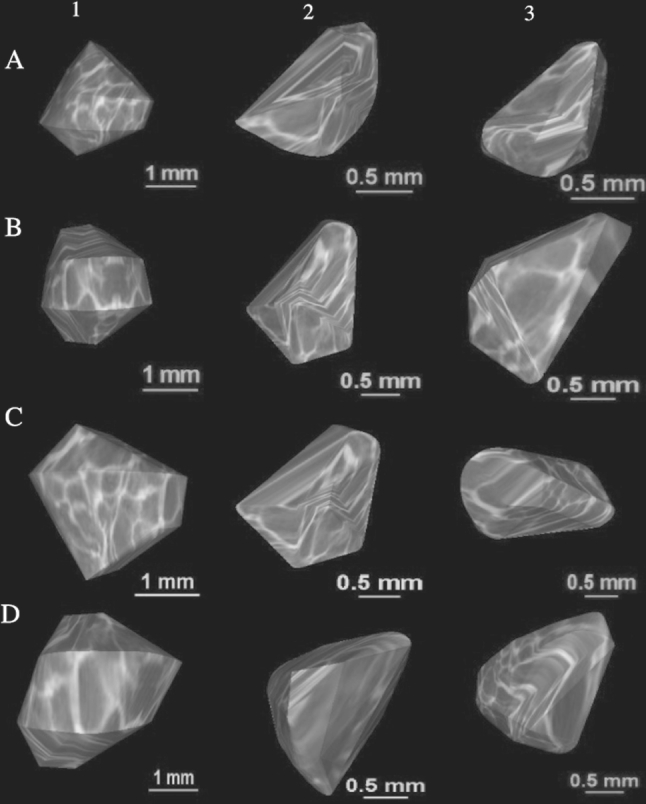
Ossification centers of the vertebral body (1), and right (2) and left (3) neural processes of vertebra C4 in fetuses aged 17 weeks (**a**), 22 weeks (**b**), 26 weeks (**c**), and 30 weeks (**d**)

**Fig. 5:**
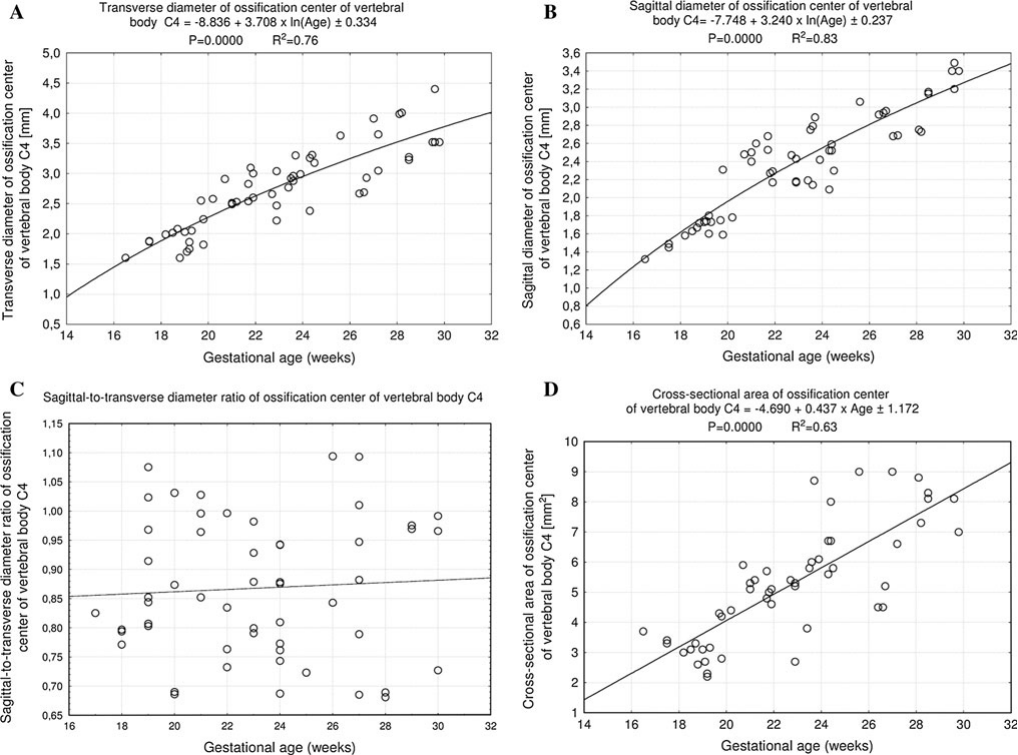
Regression lines for transverse diameter (**a**), sagittal diameter (**b**), sagittal-to-transverse diameter ratio (**c**), and cross-sectional area (**d**) of the ossification center of the vertebral body C4

**Fig. 6:**
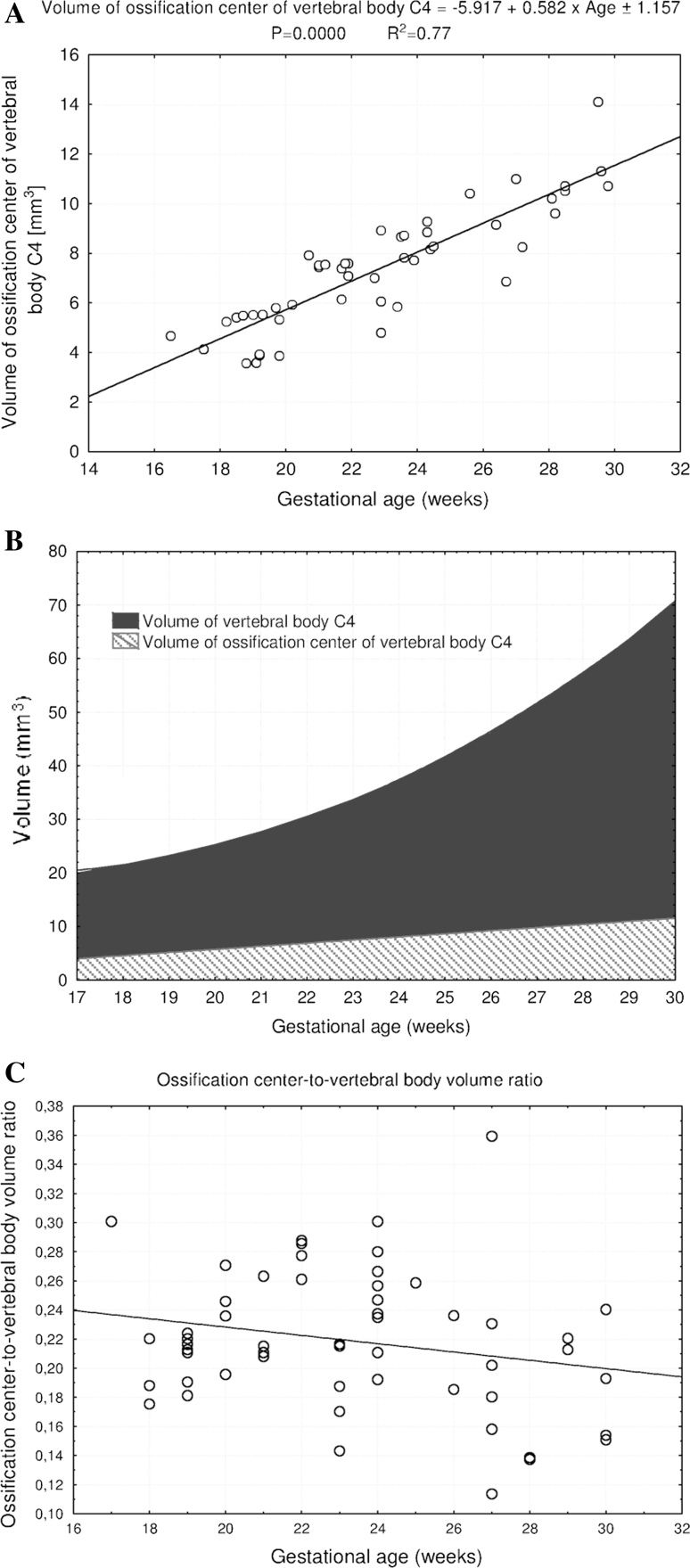
Regression lines for volume of the ossification center of the vertebral body C4 (**a**), when compared with volume of the vertebral body C4 (**b**), and the ossification center-to-vertebral body volume ratio (**c**)

The size of the ossification center of the C4 vertebral body has been presented in Table 3, while Fig. 4 presents the three ossification centers of vertebra C4 within its body (1), and right (2) and left (3) neural processes in fetuses aged 17, 22, 26, and 30 weeks, respectively. During the analyzed period, the transverse (Fig. 5a) and sagittal (Fig. 5b) diameters of the ossification center of the vertebral body grew logarithmically from 1.60 to 3.74 ± 0.44 mm, and from 1.32 to 3.37 ± 0.12 mm, according to the following models: *y* = − 8.836 + 3.708 × ln(Age) ± 0.334 (*R* ^2^ = 0.76) and *y* = − 7.748 + 3.240 × ln(Age) ± 0.237 (*R* ^2^ = 0.83), respectively. As a result, the growth dynamics for transverse and sagittal diameters decreased with gestational age, from 0.21 to 0.13 mm per week, and from 0.19 to 0.11 mm per week (*P* < 0.01), respectively. During the study period, the sagittal-to-transverse diameter ratio of the ossification center (Fig. 5c) increased from 0.86 ± 0.04 to 0.88 ± 0.11 (*P* < 0.05). The cross-sectional area of the ossification center (Fig. 5d) increased linearly from 3.70 mm^2^ in fetuses aged 17 weeks to 10.23 ± 3.14 mm^2^ in fetuses aged 30 weeks, according to the function: *y* = −4.690 + 0.437 × Age ± 1.172 (*R* ^2^ = 0.63). Similarly, the volumetric growth of the ossification center (Fig. 6a), from 4.67 to 13.45 ± 3.20 mm^3^, followed linearly as *y* = −5.917 + 0.582 × Age ± 1.157 (*R* ^2^ = 0.77).

The volumetric growth of the C4 vertebral body and its ossification center (Fig. 6b) is presented in a relative manner by the ossification center-to-vertebral body volume ratio. As shown in Fig. 6c, its value gradually decreased from 0.23 ± 0.04 to 0.21 ± 0.03 during the study period (*P* < 0.05).

**Fig. 7:**
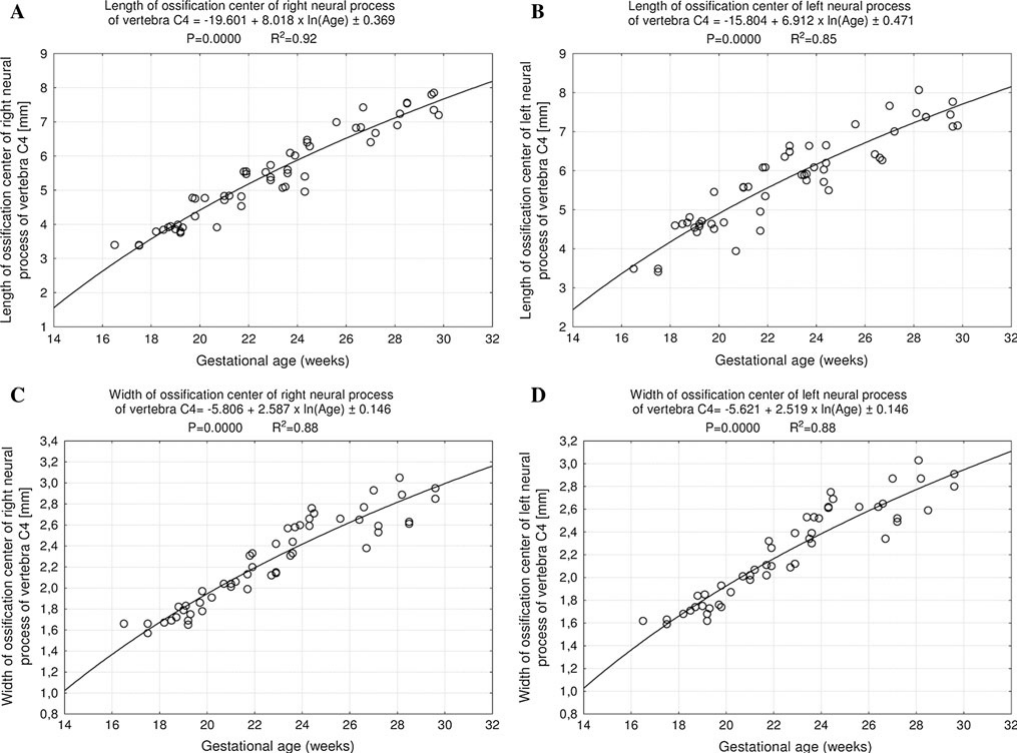
Regression lines for length on the right (**a**) and left (**b**) and for width on the right (**c**) and left (**d**) of the ossification center of the neural processes

**Fig. 8:**
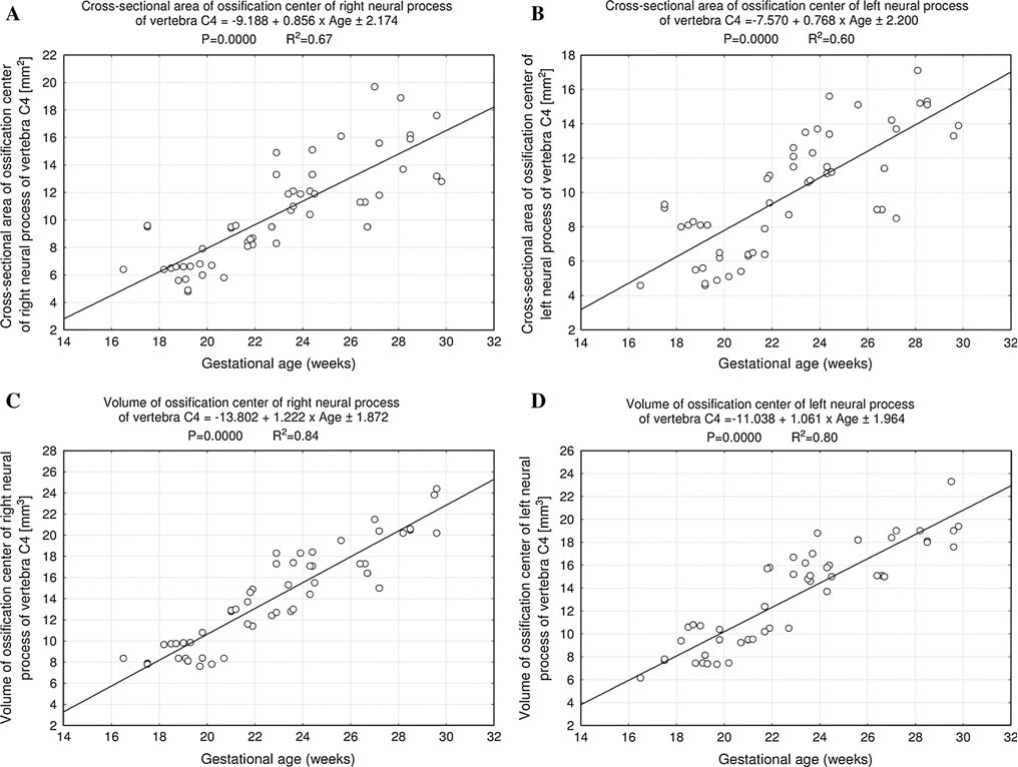
Regression lines for cross-sectional area on the right (**a**) and left (**b**) and for volume on the right (**c**) and left (**d**) of the ossification center of the neural processes

The size of ossification centers of the neural processes has been listed in Table 4. Although the right–left differences for the entire group were not statistically significant, the results have already been presented separately for each neural process, because of their great inter-individual variability. The ossification center of the neural process grew in length from 3.40 to 7.55 ± 0.32 mm on the right (Fig. 7a), and from 3.49 to 7.38 ± 0.30 mm on the left (Fig. 7b), in correspondence with the logarithmic functions: *y* = −19.601 + 8.018 × ln (Age) ± 0.369 (*R* ^2^ = 0.92) and *y* = −15.804 + 6.912 × ln(Age) ± 0.471 (*R* ^2^ = 0.85), respectively. Its width increased from 1.66 to 2.67 ± 0.27 mm on the right (Fig. 7c), and from 1.62 to 2.59 ± 0.32 mm on the left (Fig. 7d), following the logarithmic functions: *y* = −5.806 + 2.587 × ln(Age) ± 0.146 (*R* ^2^ = 0.88) and *y* = −5.621 + 2.519 × ln(Age) ± 0.146 (*R* ^2^ = 0.88), respectively. The cross-sectional area of the ossification center for the neural process revealed an increase from 6.40 to 18.05 ± 7.36 mm^2^ on the right (Fig. 8a), and from 4.60 to 15.03 ± 5.60 mm^2^ on the left (Fig. 8b), as the linear functions: *y* = −9.188 + 0.856 × Age ± 2.174 (*R* ^2^ = 0.67) and *y* = −7.570 + 0.768 × Age ± 2.200 (*R* ^2^ = 0.60), respectively. The growth in volume of the right (Fig. 8c) and left (Fig. 8d) ossification centers of the neural processes varied from 8.37 to 21.53 ± 3.15 mm^3^, and from 6.17 to 19.83 ± 2.44 mm^3^, respectively, following the linear functions: *y* = −13.802 + 1.222 × Age ± 1.872 (*R* ^2^ = 0.84), and *y* = −11.038 + 1.061 × Age + 1.964 (*R* ^2^ = 0.80).

## Discussion

The spine starts to ossify in the 8th gestational week [4] and from the 9th week it can be monitored ultrasonographically. In fetuses aged 11 weeks, ossification centers are detectable within the T2–L2 vertebral bodies and the C1–L1 neural processes [3]. Histological studies showed mineralization in much younger specimens than radiological observations [4]. The ossification timing was found to be significantly earlier in females than in males [31]. Vertebra S5 is just one example of this, because ossification centers were identified in its body and neural processes in 42.9 and 28.6 % of the females, respectively, and in no one male at the same gestational age [31]. In this aspect, our findings do not correspond with the existing literature, because no statistically significant sex differences were found in the material under examination. The possible explanation to this may be partly attributed either to the great inter-individual variability of the fetuses examined or to the different methods used.

Assessment of the fetal vertebral column in both transverse and parasagittal planes is an integral part of routine ultrasound scanning [24]. The cervical spine length has previously been reported to be linear [22], parabolic [2], or exponential [24] when related to advancing gestational age. Bagnall et al. [2] showed that in fetuses aged 8–26 weeks, the cervical spine length grew parabolically from 9 to 27 mm, being precisely expressed by the quadratic function *y* = −10.28 + 107.98 × Age − 67.35 × Age^2^ (*R* = 0.90, Age—in years) with a negative coefficient of power 2 causing a gradually decreasing growth rate. Although the entire presacral spine showed slowed down growth, both the cervical and lumbar parts slowed down to approximately half the growth rate of the thoracic part [2]. Therefore, in fetuses at the age of 8 and 26 weeks, the cervical part of the spine was about 60 % that of the thoracic part. Furthermore, the length of the “average” cervical unit (vertebra plus disc) at 26 weeks of gestation attained the value of 3.9 mm. Dimeglio et al. [9, 10] presented the longitudinal growth of the cervical spine from birth to maturity. At birth, its length measured 3.7 cm, and grew approximately by 9 cm to reach the adult size of 12–13 cm. It should be emphasized that the cervical spine doubled its length around 6 years of age and gained about 3.5 cm during the pubertal growth spurt. In our opinion, the aforementioned numerical data support that in children and adolescents, the cervical spine shows slowing down of its lengthwise growth, maybe even in a quasi-logarithmic fashion. Thus, the evolving vertebra seems to grow in the same manner both in fetuses and children. In addition, intervertebral discs accounted for the cervical spinal length, approximately 30 % at birth and 22 % at maturity [9, 10]. As reported by Tulsi [28], the heights of all cervical vertebrae continued to increase until adulthood by 39–45 % between 2–4 and 17–19 years.

In the present study, the height and transverse and sagittal diameters of the C4 vertebral body did not create linear, quadratic, or exponential functions on nomograms. In fact, we proved that the best-fit growth models were the following logarithmic functions: *y* = −3.866 + 2.225 × ln (Age) ± 0.238 for its height, *y* = −7.077 + 3.547 × ln (Age) ± 0.356 for its transverse diameter, and *y* = −3.886 + 2.272 × ln (Age) ± 0.222 for its sagittal diameter. As a consequence, their growth velocity gradually decreases with age, as previously reported by Bagnall et al. [2]. According to Tulsi [28], between 2–4 years and adulthood, the transverse and sagittal diameters increased by 6 % (6–12 %) and 33 % (20–33 %), respectively. In the material under examination, the vertebral body did not show a proportionate evolution because the sagittal-to-transverse diameter ratio declined from 0.84 ± 0.07 to 0.77 ± 0.06 during the duration of the analyzed period. Since both the transverse and sagittal diameters of the vertebral body increased logarithmically, its cross-sectional area being approximately a product of these two diameters computed the linear fashion *y* = −7.205 + 0.812 × Age ± 1.668.

The overall growth rate of the vertebral body was best expressed by measuring its volume [28]. Schild et al. [24] presented three-dimensional sonographic volume calculation of the T12–L5 vertebral bodies in fetuses aged 16–37 weeks. Their growth in volume varied in correspondence (*P* < 0.01) with exponential functions. It is noteworthy that in the material under examination, the vertebral body volume varied from 15.53 to 72.43 ± 9.46 mm^3^, with the best-fit model for volume presented by the four-degree polynomial function *y* = 14.108 + 0.00007 × Age^4^ ± 6.289. This model may probably result from multiplying the three values for height and transverse and sagittal diameters, each changing logarithmically. Postnatally, an increase in volume of the cervical vertebrae by 58–68 % was reported between 2–4 and 17–19 years, but without any regression models [28].

After reviewing the medical literature on developmental pathways of vertebral ossification centers, we failed to find any data for their dimensions [2–4, 22, 31]. Thus, the present study is the first to provide the literature with completely novel reference values and growth dynamics for length, width, cross-sectional area and volume of the three ossification centers of vertebra C4 in human fetuses. As illustrated in Tables 3, 4 and Fig. 4, the ossification center of the vertebral body offered a sharp contrast, being much larger than that of each neural process. However, it should be emphasized that the growth dynamics for all the three ossification centers of vertebra C4 were similar to each other. As a result, both their transverse and sagittal diameters increased logarithmically, while both their cross-sectional areas and volumes generated straight lines. It is important to note, however, that the sagittal-to-transverse diameter ratio of ossification center of the vertebral body was found to increase with gestational age from 0.86 ± 0.04 to 0.88 ± 0.11. It should also be noted that the vertebral body and its ossification center grew in volume according to the four-degree polynomial (*y* = 14.108 + 0.00007 × Age^4^ ± 6.289) and linear (*y* = − 5.917 + 0.582 × Age ± 1.157) functions, respectively. As a consequence, the relative size of the ossification center gradually declined with age, from 0.23 ± 0.04 at 17 weeks to 0.21 ± 0.03 at 30 weeks of gestation.

As far as the neural processes are concerned, their left and right ossification centers developed symmetrically, with no laterality differences. On the right and left sides, both their lengths (*y* = −19.601 + 8.018 × ln (Age) ± 0.369, *y* = −15.804 + 6.912 × ln(Age) ± 0.471) and widths (*y* = −5.806 + 2.587 × ln(Age) ± 0.146, *y* = −5.621 + 2.519 × ln(Age) ± 0.146) increased logarithmically. On the other hand, both their cross-sectional areas (*y* = −9.188 + 0.856 × Age ± 2.174, *y* = −7.570 + 0.768 × Age ± 2.200) and volumes (*y* = −13.802 + 1.222 × Age ± 1.872, *y* = −11.038 + 1.061 × Age + 1.964) generated straight lines. Such morphometric data have not been previously reported, thereby limiting discussion on quantitative anatomy of ossification centers. Ossification progression within the neural processes is of relevance in the diagnosis of neural tube defects [4, 11, 17, 18].

Due to age-specific reference values for vertebra C4, such abnormalities as hemivertebra, butterfly vertebra, block vertebrae, and spina bifida may ultrasonographically be diagnosed and monitored in fetuses [32]. Hemivertebra is characterized by a wedge-shaped vertebra with the absence (aplasia) of one of the two chondrification centers within the vertebral body, resulting in substantial deformity of the spine [15] in its sagittal and coronal alignment. Butterfly vertebra refers to the failure of fusion of two chondrification centers with the persistent notochord separating them [7, 23]. Both hemivertebra and butterfly vertebra may be associated with skeletal anomalies [12], diastematomyelia [20], cardiac, urogenital and gastrointestinal anomalies, and some conditions including Jarcho–Levin, Klippel-Fiel, VATER, VACTERL, and OEIS syndromes [30]. Block vertebrae are the consequence of their mal-segmentation and fusion through neighboring intervertebral discs. Spina bifida is characterized by a midline cleft between the two neural processes [5, 13, 18, 27]. Furthermore, detailed knowledge on the normal growth of spinal ossification centers in fetuses may be helpful in the prenatal diagnosis of skeletal dysplasias (osteochondrodysplasias). Such dysplasias result in both delayed ossification centers and widespread demineralization, typical of osteogenesis imperfecta type II [29], achondrogenesis [26], and thanatophoric dysplasia type I [29]. In infants with life-threatening conditions, inorganic pyrophosphate is accumulated extracellularly, resulting in both rickets and osteomalacia and finally in progressive chest and spine deformity [34].

The main limitation of this study is a relatively narrow fetal age, ranging from 17 to 30 weeks of gestation. Were we to collect a larger fetal sample with a wider age range, it would be possible to improve the growth curves obtained. Another partial limitation may be that all measurements were performed by one observer in a blind fashion. Finally, our results have been presented as if describing a sequential process in one specimen, even though the data were obtained from the cross-sectional study of 55 fetuses.

In summary, this is a cross-sectional study that describes the normative data of fetal vertebra C4 and documents its evolution. Our reference values for vertebra C4 and its three ossification centers may facilitate the diagnosis of many spinal disorders in human fetuses.

## Conclusions

1. No sex differences are found in the morphometric parameters of growing vertebra C4 and its three ossification centers.
2. The C4 vertebral body increases logarithmically in height and both sagittal and transverse diameters, linearly in cross-sectional area, and four-degree polynomially in volume.
3. The three ossification centers of vertebra C4 grow logarithmically in both transverse and sagittal diameters, and linearly in both cross-sectional area and volume.
4. The age-specific reference intervals for evolving vertebra C4 may be useful in the prenatal diagnosis of congenital spinal defects.
